# Supplementary material for: Multi-omics in nasal epithelium reveals three axes of dysregulation for asthma risk in the African Diaspora populations
Source: Nat Commun. 2024 May 28;15:4546. doi: 10.1038/s41467-024-48507-7 (PMC11133339; doi:10.1038/s41467-024-48507-7)

Supplementary Fig 1. Ancestry deconvolution in N=536 study subjects from seven CAAPA recruitment sites. **Panel A:** Global ancestry estimated using ADMIXTURE with k=3. **Panel B:** Principal components analysis (PCA) with (bottom) and without (top) reference populations. **Panel C:** Scree plot showing Eigen values of the first 22 PCs from the PCA analysis. Source data are provided as a Source Data file.

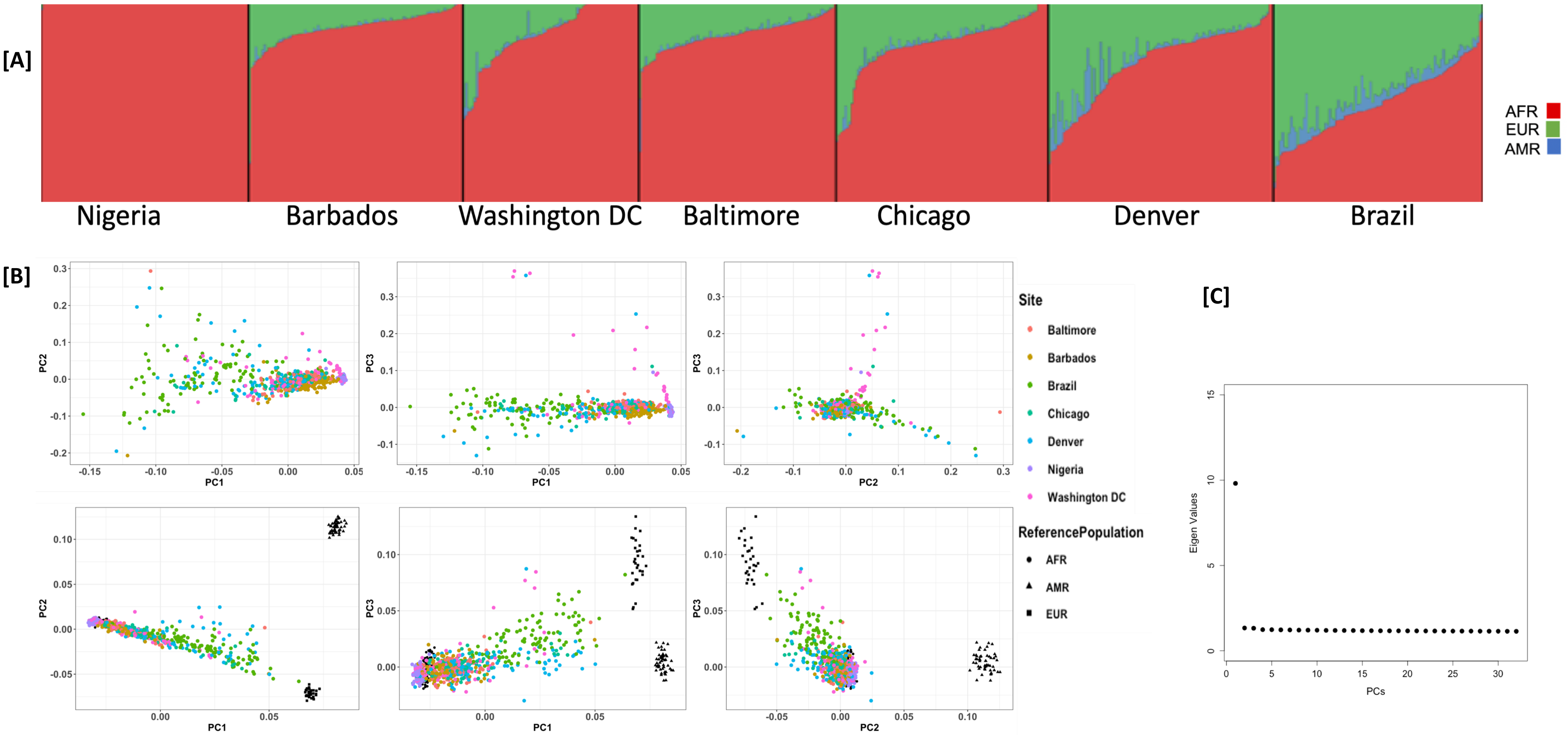

Supplementary Fig 2. DEG effect sizes for top 15 DEGs identified in the active asthma analysis for CAAPA. CAAPA sites are ordered based on average African ancestry (%YRI) from highest (Nigeria) to lowest (Brazil). Gene names are color coded based on their biological implication: purple for airway remodeling, blue for drug response, and pink for Th2 inflammation. Source data are provided as a Source Data file.

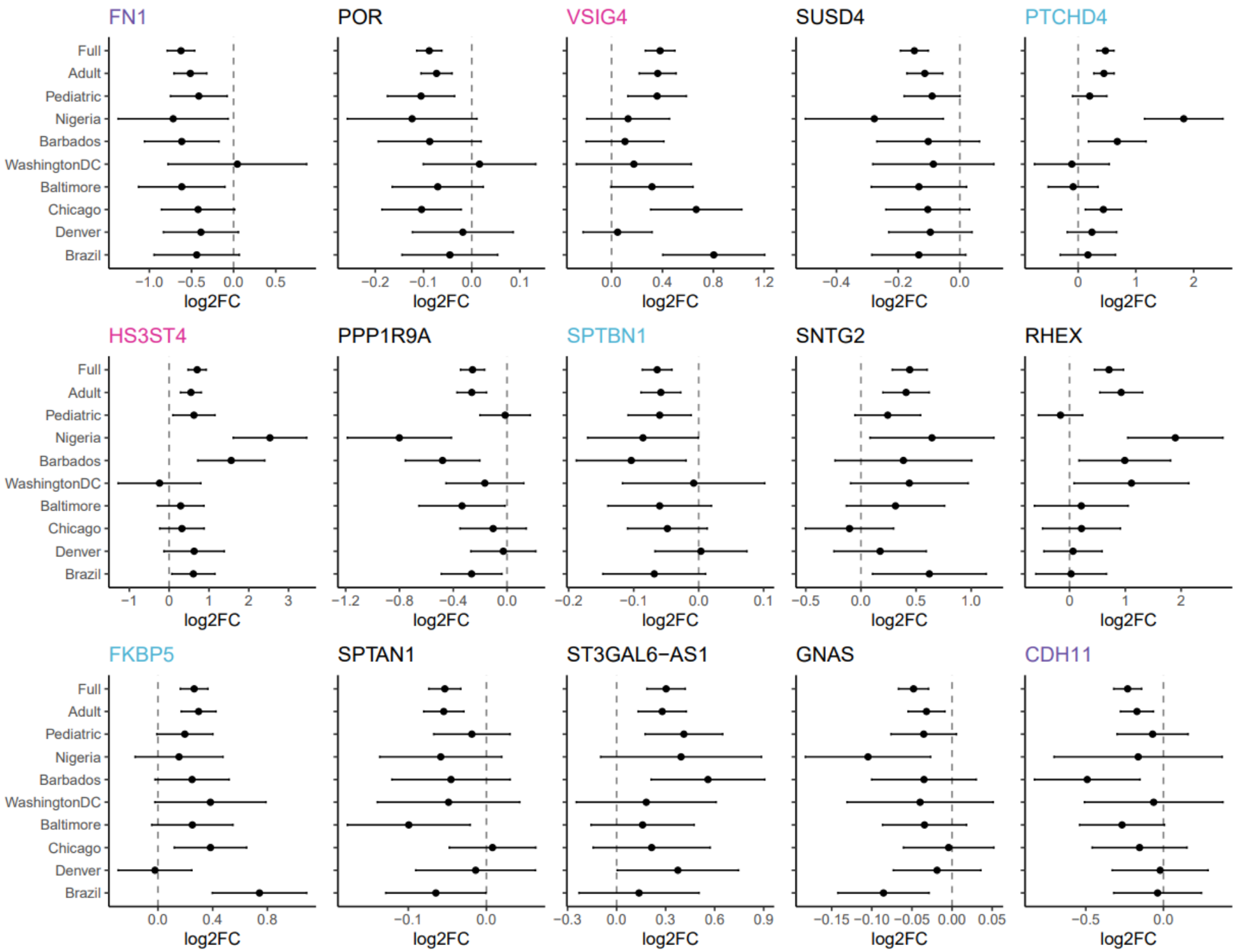

Supplementary Fig 3. Ingenuity Pathway Analysis (IPA) based on the N=389 DEGs with FDR<0.05 for active asthma. **Panel A:** IPA upstream regulator results showing p-value of overlap vs. activation z-score for all regulators where both test statistics were calculated. Activation z-scores were not calculated for regulators where the direction of change in expression was random compared to expectations derived from literature. The top 10 significant upstream regulators are labeled. P-value and Z-score significance cut offs, indicated by blue lines, were selected based on the suggested cut offs specified by IPA. **Panel B-E:** Upstream regulator networks for IL4 (B) TGFβ1 (C) and dexamethasone (C) and fluticasone (B). The color of the targets indicate log2FC in DE analysis of full dataset (Table S2). Detailed legend for panels B-E are shown in Fig S4. Source data are provided as a Source Data file.

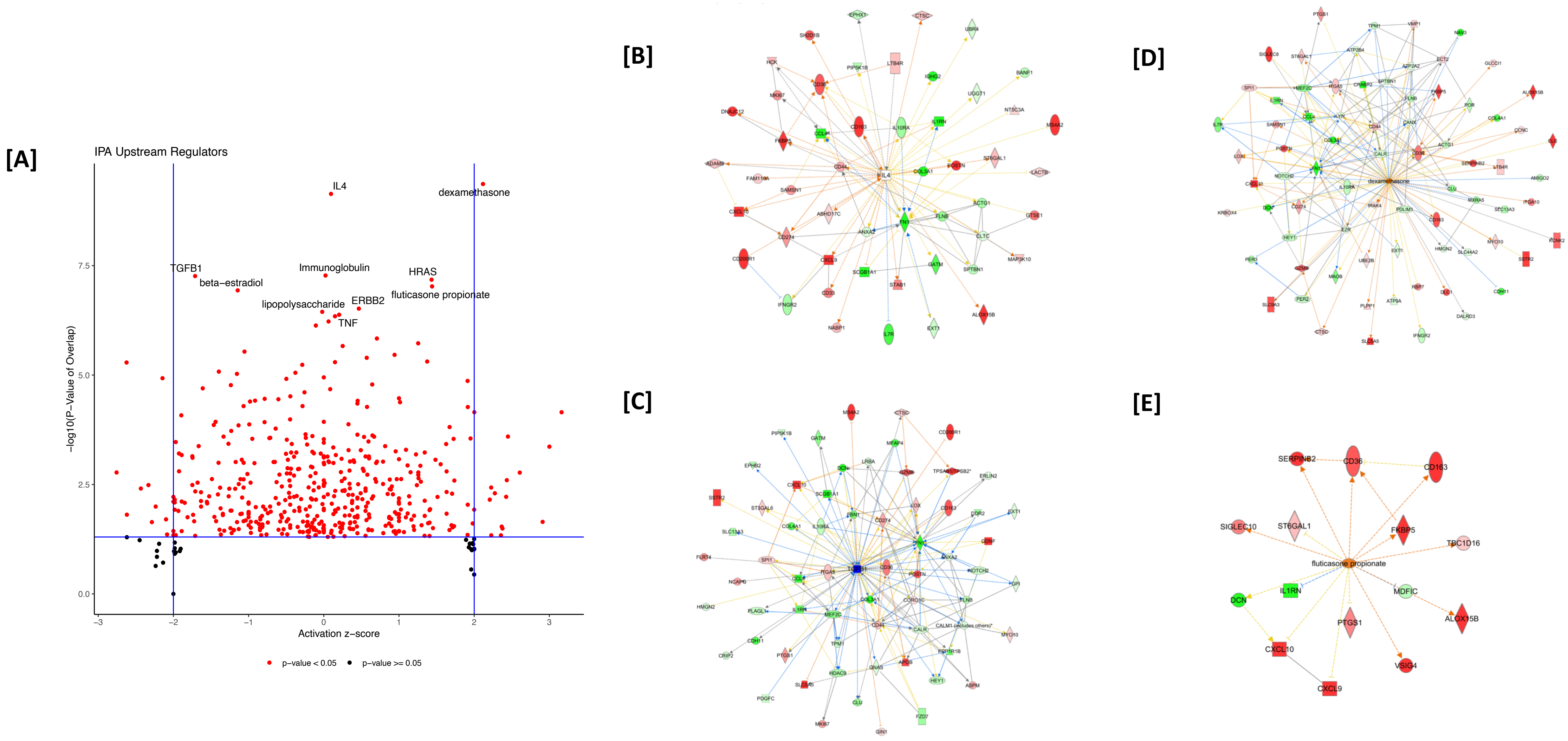

Supplementary Fig 4. Ingenuity Pathway Analysis legend for upstream regulator networks

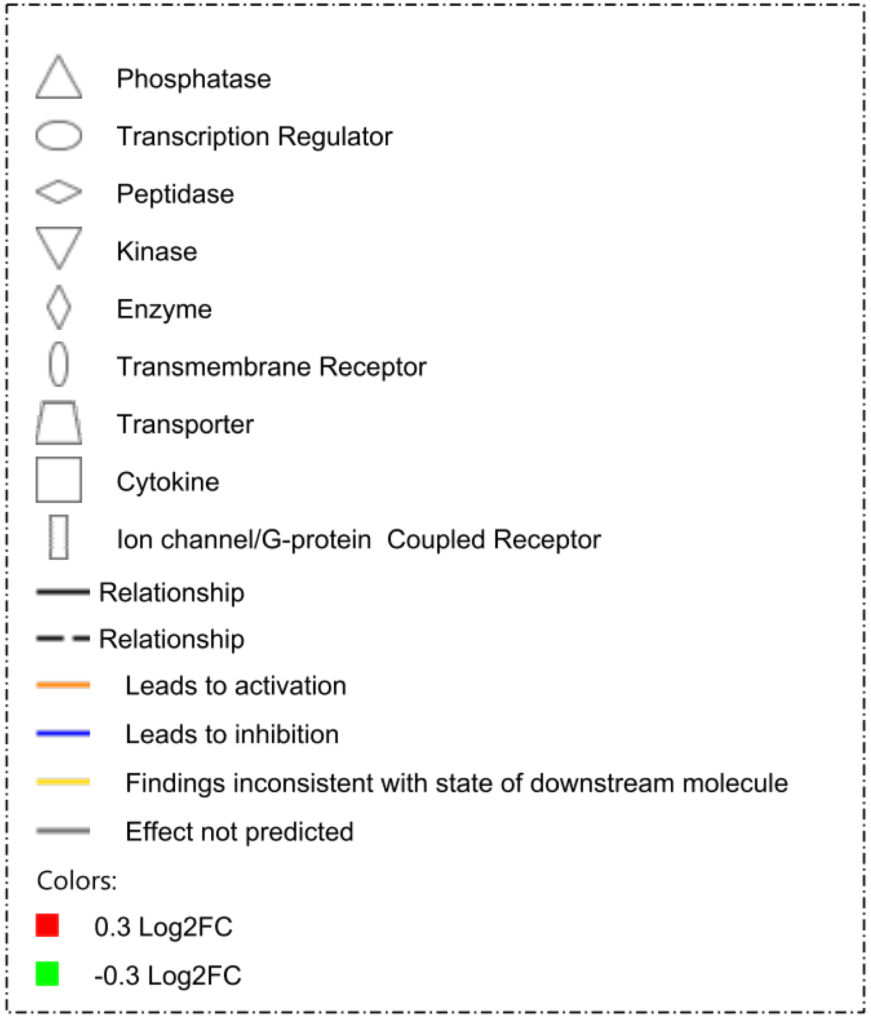

Supplementary Fig 5. STRING network retrieved for genes assigned to module M6 (CEACAM5) and M2 (CPA3). Each node represents a gene and each edge represents a protein-protein interaction with a stringdb score>0.15. Node color intensity corresponds to log2FC in DE analysis of asthma (red increased in cases compared to controls, green decreased in cases compared to controls). Node size was made proportional to the number of interactions of the node divided by maximum number of interactions of a node in the gene module (dg/max dg of module). Unconnected nodes were not included. Edge weight and transparency indicate stringdb score (wider, darker edges indicate higher score). Source data are provided as a Source Data file.

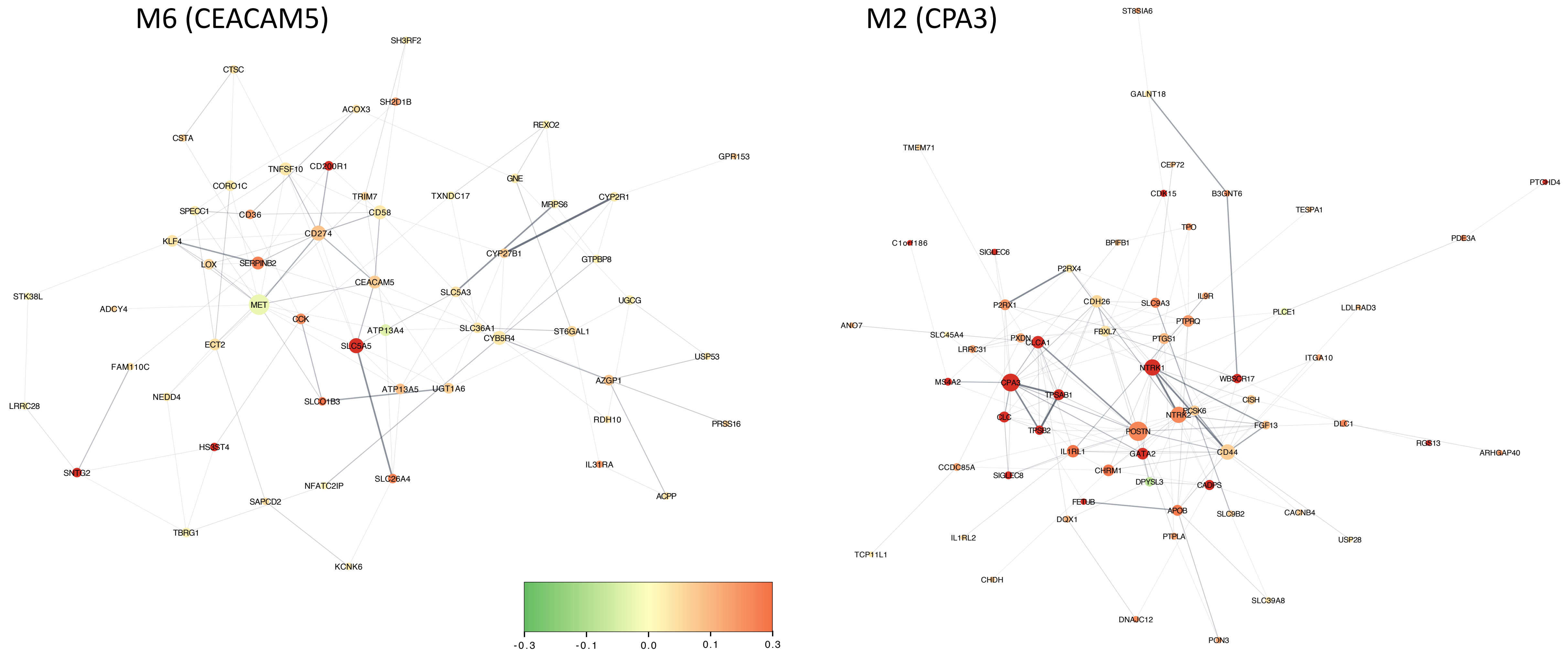

Supplementary Fig 6: Integrative analysis of methylation and gene expression at *TREML2* and *TMEM71*. **Panel A:** Distribution of gene expression and cpg methylation by asthma status and box plots showing median, lower and upper quartiles, whiskers extending to the furthest data point no more than 1.5 times the distance between the lower and upper quartiles, and outliers, by asthma case and control status for N=298 individuals. **Panel B:** Summary of association effect sizes and p-values relating gene expression, methylation and asthma. Effect sizes and unadjusted p-values from two-sided multivariate linear regression models for DMC analysis, eQTM analysis and DEG analysis pre- and post-adjustment for methylation beta at the CpG (labeled DEG_{unadj} and *DEG*, $_{adj}$), to determine if there is an association between gene expression of *FKBP5* and asthma independent of methylation. Upper row is *TREML2* and bottom row is *TMEM71*. Source data are provided as a Source Data file.

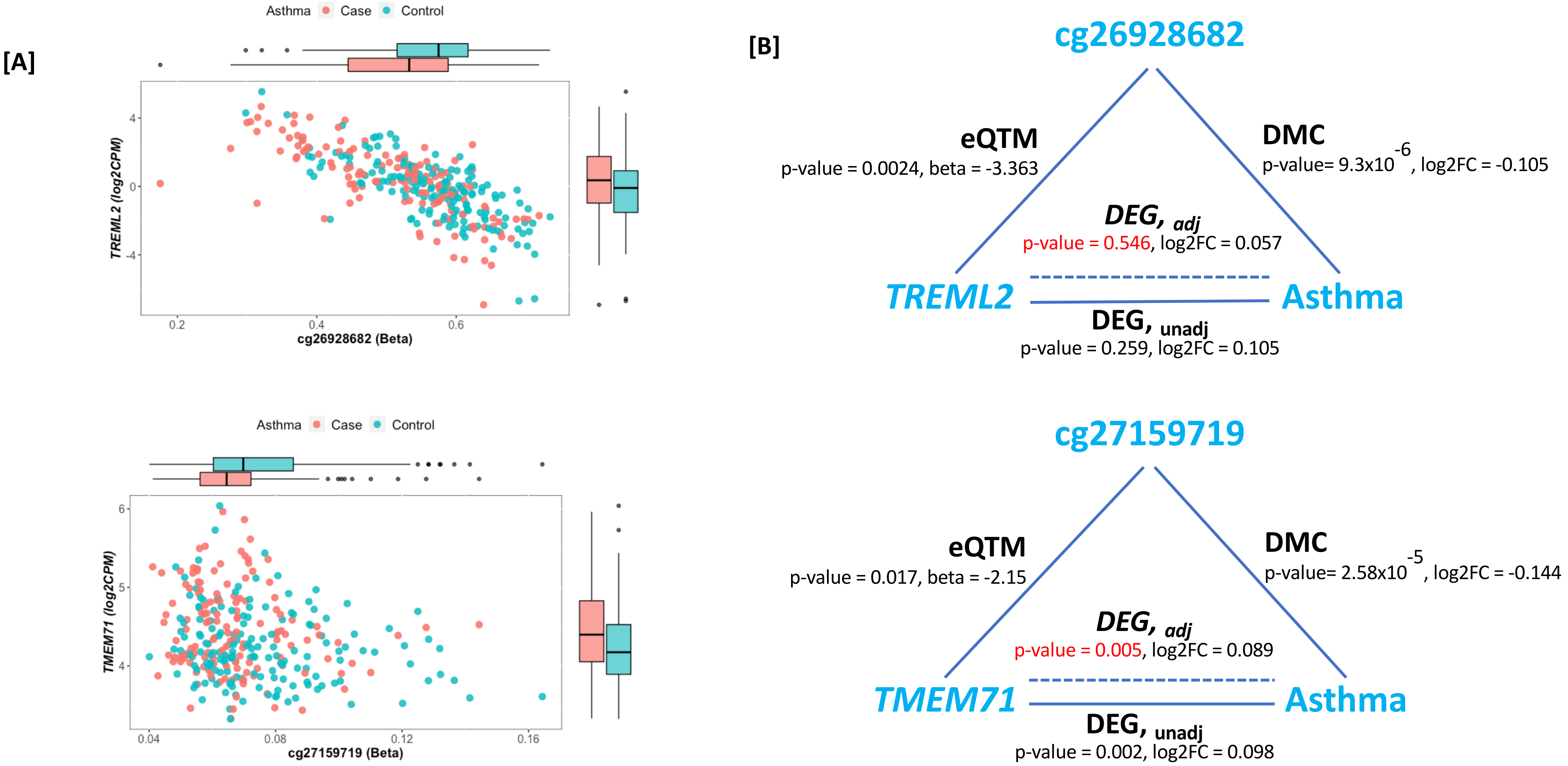

Supplement: Supplementary file 1 — Supplementary Information [file 41467_2024_48507_MOESM1_ESM.pdf]
