# Supplementary material for: Multi-omics in nasal epithelium reveals three axes of dysregulation for asthma risk in the African Diaspora populations
Source: Nat Commun. 2024 May 28;15:4546. doi: 10.1038/s41467-024-48507-7 (PMC11133339; doi:10.1038/s41467-024-48507-7)
Supplement: Supplementary file 3 — Description of Additional Supplementary Files [file 41467_2024_48507_MOESM3_ESM.pdf]

Supplementary Data Legends

File Name: Supplementary Data .xlsx
Spreadsheet: Supplementary Data 1
Description: Clinical characteristics of CAAPA active asthma cases and never-asthma controls by recruitment site.

File Name: Supplementary Data .xlsx
Spreadsheet: Supplementary Data 2
Description: DEG Results for 21,831 genes passing filtering step (CPM>0 in 47.2% of samples and mean normalized count>=20) from CAAPA multi-site analysis. Log2FC represents change in expression in active asthmatics compared to healthy controls. FDR was defined as q value given number of genes tested (i.e. q<0.05 for 2,1831 genes in full group, 21,789 genes in adults, and 21,887 genes tested for children). Replication evidence is defined as a gene previously identified to be a DEG (q<0.05) from meta-analysis in nasal epithelium with same direction direction of effect by Tsai et al. Genes with full group Q-value<0.15 were selected for WGCNA and assigned modules M1-M24. Genes with module label "M0" were unassigned. Upstream Regulators determined by IPA for genes identified as upstream regulators by IPA (p<0.05) with corresponding activation z-score when available. The test used in the DEG analysis was a moderated two-sided t-statistic.

File Name: Supplementary Data .xlsx
Spreadsheet: Supplementary Data 3
Description: 15 most significant DEGs from the CAAPA multi-site analysis. Log2FC represents change in expression in active asthmatics compared to healthy controls. FDR was defined as q value given number of genes tested (i.e. q<0.05 for 21,831 genes in full group, 21,789 genes in adults, and 21,887 genes tested for children). Genes with q<0.05 in analysis stratified on age, or p<0.05 in analysis stratified on site are highlighted with the bolded p values. Genes replicated are identified by a '*' and replication was defined as a gene previously identified to be a DEG (q<0.05) from meta-analysis in nasal epithelium with same direction direction of effect by Yang et al. The full set of genes and their replication evidence is available in Supplementary Table S2. The test used in the DEG analysis was a moderated two-sided t-statistic.

File Name: Supplementary Data .xlsx
Spreadsheet: Supplementary Data **4**
Desription: Top 10 upstream Regulators determined by IPA. Activation z-score predicts the activation state of the regulator given the direction of gene expression of gene targets in the dataset and activation/inhibition pattern given by the IPA knowledgebase network. Regulators with activation z-score> 2.0 are predicted "Activated" and regulators with with activation z-score< -2 are predicted "Inhibited". P-value of overlap measures of significance of enrichment of regulated genes in the dataset given a regulator, agnostic to direction. Upstream regulators that were present in the genes tested for differential expression are represented with an Ensembl ID. P-value of overlap is based on the one-sided fishers exact test.

File Name: Supplementary Data .xlsx
Spreadsheet: Supplementary Data **5**

Description: DEG anlaysis for 188 genes identified as direct or indirect targets of Dexamethasone and Fluticasone by Ingenuity Pathway Analysis. Medications use of 253 active asthmatics was defined by CASI question 4b: score 0-1 no treatment/albuterol as needed (N=154) and score 2-5 low-high dose ICS (N=99). log2FC represents change in expression comparing asthmatics using low-high dose ICS (n=99) to asthmatics with no treatement/albuterol as needed(n=154). No gene is significant at the Bonferroni threshold (p<0.05/188)**.** Gene targets of upstream regulators with known relationships derived from literature compiled in the Ingenuity Knowledge Base are indicated as "direct" targets. Gene targets predicted in the mechanistic network of a regulator, connected to the upstream regulator through other upstream regulators, are indicated as "indirect". The test used in the DEG analysis was a moderated two-sided t-statistic.

File Name: Supplementary Data .xlsx
Spreadsheet: Supplementary Data **6**
Description: DEG Results for 1,326 genes passing with FDR<0.15 for the full asthma group (subset from **Supplementary Table 2**) and for the comparison of asthma cases on nasal steroids (NS) that did not withhold NS usage 5 days prior to nasal epithelium sampling (N=32) vs. those were able to whithold NS use (N=36). FDR for the NS analysis was evalulated for 1,326 genes. The test used in the DEG analysis was a moderated two-sided t-statistic.
File Name: Supplementary Data .xlsxSpreadsheet: Supplementary Data **7**
Description: Significant eQTM results for DEGs paired with CpGs within 5kb of the transcription start site (TSS) or linked to the gene by promoter-capture HiC experiments in bronchial epithelial cells. There are N=915 unique CpGs identified from the N=918 eQTMs that were moved forward to DMC analysis. Effect sizes (beta and log2 fold-change) and unadjusted p-values are from two-sided multivariate linear regression models. Significance for eQTMs was evaluated as uncorrected p<0.05. Significance for DMCs was evaluated at the Bonferroni threshold of p<0.05/915. DEGs are as described in **Supplementary Table 2** with FDR (q-value) <0.05. Table is sorted on DMC p-value, and DMCs with Bonferroni corrected significance marked in bold.

File Name: Supplementary Data .xlsx
Spreadsheet: Supplementary Data **7**
Description: Significant eQTM results for DEGs paired with CpGs within 5kb of the transcription start site (TSS) or linked to the gene by promoter-capture HiC experiments in bronchial epithelial cells. There are N=915 unique CpGs identified from the N=918 eQTMs that were moved forward to DMC analysis. Effect sizes (beta and log2 fold-change) and unadjusted p-values are from two-sided multivariate linear regression models. Significance for eQTMs was evaluated as uncorrected p<0.05. Significance for DMCs was evaluated at the Bonferroni threshold of p<0.05/915. DEGs are as described in Supplementary Data 2 with FDR (q-value) <0.05. Table is sorted on DMC p-value, and DMCs with Bonferroni corrected significance marked in bold.

File Name: Supplementary Data .xlsx
Spreadsheet: Supplementary Data **8**

Description: Significant DMCs (Bonferroni-corrected p-value < 0.05/915 for the 915 unique CpGs from eQTM results with p<0.05, see **Supplementary Table 7**) for N=389 DEGs paired with CpGs within 5kb of the transcription start site (TSS) or linked to the gene by promoter-capture HiC (pcHiC) experiments in bronchial epithelium. For *FKBP5* and *TREML2* with multiple identified eQTLs, independance of the CpGs was tested in a joint model including both CpGs from the independent models and the sentinel CpG is identified in bold font. All analysis was limited to the subjects from the four US recruitment sites. Effect sizes (beta) and unadjusted p-values are from two-sided multivariate linear regression models.
