# Supplementary material for: Multi-omics in nasal epithelium reveals three axes of dysregulation for asthma risk in the African Diaspora populations
Source: Nat Commun. 2024 May 28;15:4546. doi: 10.1038/s41467-024-48507-7 (PMC11133339; doi:10.1038/s41467-024-48507-7)
Supplement: Supplementary file 5 — Reporting Summary [file 41467_2024_48507_MOESM5_ESM.pdf]

nature portfolio

Corresponding author(s): Rasika A Mathias

Last updated by author(s): 06-05-23

Reporting Summary

Nature Portfolio wishes to improve the reproducibility of the work that we publish. This form provides structure for consistency and transparency in reporting. For further information on Nature Portfolio policies, see our Editorial Policies and the Editorial Policy Checklist.

Statistics

For all statistical analyses, confirm that the following items are present in the figure legend, table legend, main text, or Methods section.

n/a	Confirmed	
☐	☒	The exact sample size (*n*) for each experimental group/condition, given as a discrete number and unit of measurement
☐	☒	A statement on whether measurements were taken from distinct samples or whether the same sample was measured repeatedly
☐	☒	The statistical test(s) used AND whether they are one- or two-sided<br>*Only common tests should be described solely by name; describe more complex techniques in the Methods section.*
☐	☒	A description of all covariates tested
☐	☒	A description of any assumptions or corrections, such as tests of normality and adjustment for multiple comparisons
☐	☒	A full description of the statistical parameters including central tendency (e.g. means) or other basic estimates (e.g. regression coefficient) AND variation (e.g. standard deviation) or associated estimates of uncertainty (e.g. confidence intervals)
☐	☒	For null hypothesis testing, the test statistic (e.g. *F*, *t*, *r*) with confidence intervals, effect sizes, degrees of freedom and *P* value noted<br>*Give P values as exact values whenever suitable.*
☒	☐	For Bayesian analysis, information on the choice of priors and Markov chain Monte Carlo settings
☒	☐	For hierarchical and complex designs, identification of the appropriate level for tests and full reporting of outcomes
☐	☒	Estimates of effect sizes (e.g. Cohen's *d*, Pearson's *r*), indicating how they were calculated

Our web collection on statistics for biologists contains articles on many of the points above.

Software and code

Policy information about availability of computer code

Data collection	None used
Data analysis	FastQC<br>HISAT2<br>CoCo<br>Picard version: 2.25.6<br>BBDuk Version 38.90<br>DESeq2_1.34.0<br>sva_3.42.0<br>WGCNA_1.71<br>limma_3.50.3<br>edgeR_3.36.0<br>Ingenuity Pathway Analysis 22.0.2<br>Cytoscape 3.9.1<br>StringApp 1.7.1<br>CorrConf 2.2<br>Matrix eQTL 2.3<br>peer 1.3

For manuscripts utilizing custom algorithms or software that are central to the research but not yet described in published literature, software must be made available to editors and reviewers. We strongly encourage code deposition in a community repository (e.g. GitHub). See the Nature Portfolio guidelines for submitting code & software for further information.

Data

Policy information about availability of data

All manuscripts must include a data availability statement. This statement should provide the following information, where applicable:

- Accession codes, unique identifiers, or web links for publicly available datasets
- A description of any restrictions on data availability
- For clinical datasets or third party data, please ensure that the statement adheres to our policy

The RNASeq data generated in this study have been deposited in the GEO database under accession code GSE240567 [https://www.ncbi.nlm.nih.gov/geo/query/acc.cgi?acc=GSM7701971]. The Methylation data generated in this study have been deposited in the GEO database under accession code GSE250513 [https://0-www-ncbi-nlm-nih-gov.brum.beds.ac.uk/geo/query/acc.cgi?acc=GSE250513].
Source data are provided with this paper for all figures.

Research involving human participants, their data, or biological material

Policy information about studies with human participants or human data. See also policy information about sex, gender (identity/presentation), and sexual orientation and race, ethnicity and racism.

Reporting on sex and gender	CAAPA includes both males and females, and we use the term sex in tables and methods. Sex as a biological variable is used as a covariate in analysis, no stratified analysis were performed. We do not evaluate sex-stratified effects in our multi-omics analysis of the nasal epithelium for asthma.
Reporting on race, ethnicity, or other socially relevant groupings	All study subjects are of African ancestry as this is a Consortium on Asthma among African-ancestry Populations in the Americas. Subjects are stratified on recruitment site, and we use genetic ancestry deconvolution to determine global admixture proportions and to adjust for global ancestry.
Population characteristics	The primary characteristics used in our analyses are: asthma status, age, sex, library preparation batch, site, and genetic ancestry principal components. Additional population characteristics examined include geographic sampling site, and phenotypes for medication use, total serum IgE, phadiotop, eosinophils, asthma severity, and pulmonary lung function.
Recruitment	Study subjects included African ancestry individuals recruited from three non-US sites (Nigeria, Barbados and Salvador, Brazil) and four US sites (Denver, Baltimore, Washington DC and Chicago). At all sites, cases were first defined as subjects with 'ever' asthma confirmed by a physician, and then further restricted to the subset of individuals with 'current' asthma. Controls were defined as subjects with no history of asthma. All definitions were identical between sites and on the basis on questionnaire data. There is one site where we anticipate a bias in the severity of asthma selected and this is Brazil where cases and controls were recruited through the Program for Control of Asthma in Bahia (ProAR) Severe Asthma Cohort. To address this, we randomized by site, case status, age (adult/pediatric), and sex. Additionally, all analysis was also performed stratified by site and site-specific differences were examined, and analysis accounting for medication was also performed.
Ethics oversight	All samples used for this study were obtained following written informed consent from participants. All samples used for this study were obtained following written informed consent from participants. The University of Colorado (IRB#: 17-1807), Johns Hopkins University (IRB00179053), University of Chicago (IRB18-0466-CR001), National Institutes of Health (IRB#: P184385), University of West Indies (IRB#: 190604-A), University of Bahia (IRB#: 3.302.487) and University of Ibadan Institutional Review Boards approved the conduct of this study (IRB18-0840).

Note that full information on the approval of the study protocol must also be provided in the manuscript.

Field-specific reporting

Please select the one below that is the best fit for your research. If you are not sure, read the appropriate sections before making your selection.

☒ Life sciences ☐ Behavioural & social sciences ☐ Ecological, evolutionary & environmental sciences

For a reference copy of the document with all sections, see nature.com/documents/nr-reporting-summary-flat.pdf

Life sciences study design

All studies must disclose on these points even when the disclosure is negative.

Sample size	N=536 in final included sample. The sample size was original determined on the basis of eQTLs for which an N=700 was determined to achieve 80% power at effect sizes between 0.25 and 0.5 for the eQTL effect with minor allele frequencies >5%. Here, we are leveraging the RNASeq data generated in CAAPA to perform DEG analysis.
Data exclusions	Genetics: Quality control procedures were performed on genotype data to exclude any samples or variants that had missingness >3%; standard QC steps included: sex verification, heterozygosity checks and identity-by-descent (IBD) to look for any unexpected relatedness. Two individuals failed on call rate and four individuals were identified as sex mismatches in one or more omics data sets (dropped from all datasets). Thirteen individuals showed cryptic relatedness, as being part of a parent offspring pair, full sibs or half sibs pairs based on the IBD estimates; one independent subject was selected prioritizing case status from each relationship resulting in 7 individuals being dropped from

all datasets. One sample was identified as a sample swap, duplicating another individual, and was dropped. A total of 14 samples were excluded and further ancestry analysis was limited to 673 individuals from all seven sites.

RNASeq: Four samples were excluded due to sex mismatches, 3 samples were excluded due to unexpected relatedness, 4 samples were excluded due to failure of library preparation, and 7 samples were excluded due a high percentage of ribosomal RNA.

Methylation EPIC array: Samples were excluded based on the following metrics: 12 with mean of methylated and unmethylated signal < 10.5; four with discordant sex.

Replication	For the 21,831 genes tested in the DEG analysis of all subjects, we searched for replication in a meta-analysis study of airway epithelium gene expression in asthma. Briefly, Tsai et al performed a meta-analysis of eight independent gene expression studies including both nasal and bronchial epithelium tissue. Full results from the meta-analysis were obtained from these authors and were compared to the CAAPA results by matching genes on Ensembl ID. Ensembl IDs were retrieved for the meta-analysis gene symbols from Ensembl Release 109 homo sapiens GRCh38 using pyensembl [https://github.com/openvax/pyensembl]. Each gene symbol in an observation was queried individually for matching Ensembl IDs and matched to Ensembl IDs in CAAPA. Where multiple observations in the meta-analysis matched an Ensembl ID in CAAPA, the observation with the highest number of studies (k) in the meta-analysis was selected. Enrichment of CAAPA DEGs in the meta-analysis DEGs was tested using a hypergeometric test. The total number of genes tested was determined as the number of unique Ensemble IDs retrieved in the meta-analysis full results that matched to Ensembl IDs included in the 21831 genes tested in CAAPA.
Randomization	Each batch of RNASeq and each plate for methylation was randomized with respect to case/control status, sex, adult/pediatric and geographic site.
Blinding	Blinding is not applicable for this study because it is not a clinical trial nor is there any intervention

Reporting for specific materials, systems and methods

We require information from authors about some types of materials, experimental systems and methods used in many studies. Here, indicate whether each material, system or method listed is relevant to your study. If you are not sure if a list item applies to your research, read the appropriate section before selecting a response.

Materials & experimental systems

n/a	Involved in the study
☒	☐ Antibodies
☒	☐ Eukaryotic cell lines
☒	☐ Palaeontology and archaeology
☒	☐ Animals and other organisms
☒	☐ Clinical data
☒	☐ Dual use research of concern
☒	☐ Plants

Methods

n/a	Involved in the study
☒	☐ ChIP-seq
☒	☐ Flow cytometry
☒	☐ MRI-based neuroimaging
